# Exogenous Calcium Reinforces Photosynthetic Pigment Content and Osmolyte, Enzymatic, and Non-Enzymatic Antioxidants Abundance and Alleviates Salt Stress in Bread Wheat

**DOI:** 10.3390/plants12071532

**Published:** 2023-04-01

**Authors:** Mervat Sh Sadak, Rania S. Hanafy, Fatma M. A. M. Elkady, Asmaa M. Mogazy, Magdi T. Abdelhamid

**Affiliations:** 1 Botany Department, National Research Centre, 33 El Buhouth Street, Dokki, Cairo 12622, Egypt; 2 Biological and Geological Sciences Department, Faculty of Education, Ain Shams University, Cairo 11575, Egypt; 3 Department of Soil and Crop Sciences, Texas A&M University, 370 Olsen Blvd., College Station, TX 77843, USA

**Keywords:** antioxidant, Ca^2+^, enzymes, osmoprotectant, reactive oxygen species, salinity, signal molecule, *Triticum aestivum*

## Abstract

One of the main environmental stresses that hinder crop development as well as yield is salt stress, while the use of signal molecules such as calcium (Ca) has a substantial impact on reducing the detrimental effects of salt on different crop types. Therefore, a factorial pot experiment in a completely randomized design was conducted to examine the beneficial role of Ca (0, 2.5, and 5 mM) in promoting the physiological, biochemical, and growth traits of the wheat plant under three salt conditions viz. 0, 30, and 60 mM NaCl. Foliar application of Ca increased the growth of salt-stressed wheat plants through increasing photosynthetic pigments, IAA, proline, and total soluble sugars contents and improving antioxidant enzymes in addition to non-enzymatic antioxidants glutathione, phenol and flavonoids, β-carotene, and lycopene contents, thus causing decreases in the over-accumulation of free radicals (ROS). The application of Ca increased the activity of antioxidant enzymes in wheat plants such as superoxide dismutase (SOD), peroxidase (POD), and catalase (CAT), which scavenge reactive oxygen species (ROS) and relieved salt stress. An additional salt tolerance mechanism by Ca increases the non-antioxidant activity of plants by accumulating osmolytes such as free amino acids, proline, and total soluble sugar, which maintain the osmotic adjustment of plants under salinity stress. Exogenous Ca application is a successful method for increasing wheat plants’ ability to withstand salt stress, and it has a considerable impact on the growth of wheat under salt stress.

## 1. Introduction

Wheat is regarded as a significant cereal all over the world; wheat ranks second to rice considering the intake while being the third highest cultivated cereal after maize and rice [1]. Bread wheat (*Triticum aestivum*) is moderately tolerant to salt, while durum wheat (*Triticum turgidum* ssp. durum) is less so [2].

The plant may be subject to different abiotic stress in its natural habitat, including drought, flood, salt, and cold. These unfavorable conditions prevent the growth and production of plants everywhere in the world [3]. One of those serious problems worldwide is that salt-contaminated lands in arid and semi-arid climates are greatly susceptible to the effects of climate changes on rising soil salinity [4]. Greater than 954 million hectares of the world’s agricultural area (about 20%) has high salt content [5]. The major reason for increasing salt stress in agricultural soil is irrigation with poor saline water, poor drainage, the practice of irrigation, high transpiration, and low rainfall. By the year 2050, it has been predicted that more than 50% of the arable agricultural land might be affected by salinity. High salt levels decrease the growth and yield of different crops in various ways [6]. Salinity affects plants in two ways: osmotic stress from declining soil water supply and an imbalance of solutes in the cytosol. High salt levels reduce plant ability to absorb water and minerals such as potassium (K^+^) and calcium (Ca^2+^). Increased levels of sodium (Na^+^) and chloride (Cl^−^) have an adverse effect on cell membranes and cytosol metabolism, as well as having a direct impact on plant cells [7]. The primary effect of salt results in a number of secondary stimuli, including reduced cell expansion, assimilate synthesis, reduced membrane function and cytosolic metabolism, and increased free radical production (ROS) [8].

Plants must recognize the type and intensity of external stresses, and convert those signals into an intracellular signal to trigger the necessary physiological reactions to remain alive [9]. All living organisms begin their adaptation to abiotic challenges with the sensing of this stress and the subsequent transmission of stress signals [8]. Therefore, a crucial and essential physiological problem is how plants detect abiotic stress signals, translate them into cellular signaling, and then adapt to hostile environments. To detect exterior environmental variations, a primary stress sensor is generally needed; in response to those variations, the sensor modifies signal transmission pathways and starts the proper reactions, which enable the plant to acclimatize to the stress state. Therefore, the first stage in the transfer of exterior stimuli into cellular signals by plants is the observation of the exterior environment by a sensor [10].

Plants use Ca^2+^ as a fundamental second messenger to adjust a complex network of signaling pathways to adapt to a variety of abiotic stresses [10,11]. The increased levels of intracellular Ca^2+^ are one of the primary signaling responses when plants are exposed to abiotic stimuli; thus, the subsequent Ca^2+^ signaling controls various metabolic processes in plants, such as transcriptional control and physiological subsequent developmental responses [12]. The outcomes of this study imply that Ca^2+^ is a crucial second messenger for comprehending the interactions between plants and abiotic stimuli.

Plants use multiple strategies to minimize the negative effect of salt stress and improve plant tolerance. Sodium (Na^+^) influx is derived via voltage-insensitive monovalent-cation channels (VIC). These channels are controlled by bivalent cations such as Ca^2+^ to maintain ion hemostasis. Plants have evolved the ability to detoxify excess Na^+^ amounts that accompany high salt amounts [13]. Furthermore, plants utilize calcium, a tiny and versatile element, in a variety of physiological processes [14]. Calcium is a crucial mineral for plant growth and development since it helps in many vital processes such as maintaining the integrity of the membrane and cell walls, increasing a large number of energetic enzyme activities, and interacting with phytohormones. Calcium is involved in the regulatory systems that plants trigger for adjusting salinity negative effects [15]. Calcium gains a crucial impact under salt conditions by providing stress protection via the regulation of the majority of cell wall physiological processes [16]. Stress tends to trigger several defensive responses, and calcium signaling is necessary for the development of salt tolerance [13]. Na^+^ absorption is inhibited by Ca^2+^, which lessens the effect of salt reduction on plants. Moreover, Ca^2+^ can reduce K^+^ efflux from a plant cell to lessen the harmful effects of chloride salts on plant growth [17]. Therefore, using CaCO_3_ as an exogenous treatment on different plants as a method for improving plant tolerance towards stress such as salt stress is very important.

Hence, the current study intends to explore the physiological role of calcium in reducing the negative effects of salinity on wheat plants, keeping in mind the major positive impact of calcium in reducing the adverse effects of salt stress on the wheat plant.

## 2. Materials and Methods

### 2.1. Experimental Procedure

In the winter season of 2017/2018, a pot study was set up in the National Research Centre’s greenhouse, Dokki, Cairo, Egypt, to explore the impact of CaCO_3_ foliar spraying on wheat plants irrigated with saline water. Across the study, the minimum and maximum temperatures during the day were 17.8 and 28.4 °C, respectively, while the minimum and maximum temperatures during nighttime were 8.7 and 16.7 °C, respectively. The mean daytime temperature was 23.1 ± 2.8 °C. Daily relative humidity ranged from 22.1 to 59.2%.

Grains of wheat (*Triticum aestivum* L.) var. Misr 1 were chosen, which were the same size and color. Wheat grains were sterilized for nearly 2 min with 1% sodium hypochlorite after being gently washed with distilled water. Ten identical air-dried grains were planted in plastic pots at thirty mm depth, in approximately seven kg of clay soil. Each pot was enclosed with a polyethylene bag to stop water from evaporation. This experimental soil has the following physical and chemical properties: The soil had a clay-loam texture, with coarse sand 1.4%, fine sand 31.7%, silt 39.6%, and clay 27.3%, EC_e_ 1.82 dS m^−1^, pH 7.5, organic matter 1.93%, CaCO_3_ 7.88%, and available N, P, and K accounting for 45.6, 7.8, and 415.0 mg kg^−1^, respectively. For reducing compaction and encouraging drainage, clay soil was homogenized with sand in a 3:1 ratio (*v*:*v*). The soil was fertilized with fertilizers three days before planting in the following doses: (1) ammonium sulfate (20.5% N) at 800 kg ha^−1^ dose; (2) super phosphate (15% P_2_O_5_) at 240 kg ha^−1^ dose; and (3) potassium sulfate (48% K_2_O) at a 120 kg ha^−1^ dose. Before planting, the N, P, and K fertilizers were thoroughly incorporated into each pot. A soil water holding capacity of 0.36, was determined by saturating the soil in each pot with water, letting it drain for 48 h, and weighing it. The water holding capacity was maintained at about 90% of its maximum level. Following the guidelines provided by the Egyptian Ministry of Agriculture and Land Reclamation, all recommendations and practices related to wheat production were followed.

The experiment was factorial in a completely randomized design and replicated three times. The experiment comprised two factors. The first factor included three saline water levels namely 0.0, 30, or 60 mmol/L (mM) sodium chloride (NaCl). The second factor involved three levels of calcium carbonate viz 0, 2.5, and 5 mM. Therefore, the experiment consisted of nine treatments as combinations of three salt irrigation regimens (NaCl) and three Ca^2+^ levels. NaCl and Ca^2+^ were purchased from Sigma–Aldrich, Saint Louis, MO, USA. After plant emergence, wheat seedlings were thinned ten days after sowing (DAS), and four plants per pot were left. Pots were irrigated with distilled water supplemented with 0.0, 30, or 60 mM NaCl starting from 15 DAS and lasted till the harvesting of wheat plants at 75 DAS. Spraying wheat plants with Ca^2+^ was performed at 42 and 56 DAS, coinciding with stem elongation or jointing stage GS31: First node detectable and GS39: Flag leaf fully unrolled, ligule just visible, respectively. All spray treatments were completed early morning, before 9:00 a.m., with a hand sprayer at sufficient pressure to keep droplet size small. To obtain proper coverage, plants were sprayed from all sides. The spray volume amount of water consisted of approximately 500 L per ha based on each pot area being 0.049 m^2^. Plants were sprayed from both sides of the row to achieve adequate coverage.

### 2.2. Measurements

After 75 DAS, plant samples were taken to assess growth criteria i.e., shoot length (cm), leaf area per plant (cm^2^), and plant dry weight (g plant^−1^). Additionally, some chemical analyses were performed.

#### 2.2.1. Biochemical Analysis

Photosynthetic pigments in fresh leaf tissue were determined using the method of Lichtenthaler and Buschmann [18]. Indole acetic acid content (IAA) was measured according to the method described by Gusmiaty et al. [19]. Free amino acid and proline contents were extracted according to the method of Kalsoom et al. [20]. The free amino acids were determined with the method of Tamayo and Pedrol [21]. Proline was assayed according to the method described by Versluses [22]. The total soluble carbohydrates (TSS) were determined by the method given by Chow and Landhausser [23]. Determination of total carbohydrates was carried out according to Albalasmeh et al. [24]. Hydrogen peroxide (H_2_O_2_) concentration was determined according to Velikova et al. [25]. The levels of lipid peroxidation were measured by determining the levels of malondialdehyde (MDA). Malondialdehyde is a product of lipid peroxidation and was assayed by using thiobarbituric acid reactive substrate (TBARS) contents with the method of Hodges et al. [26], with some modifications.

#### 2.2.2. Estimation of Enzyme Activities

Enzyme extractions were collected following the method described by Chen and Wang [27]. An assay of superoxide dismutase was carried out according to the method of Chen and Wang [27]. Peroxidase (POX, EC 1.11.1.7) activity was assayed by the method of Bergmeyer [28]. The catalase activity (CAT, EC 1.11.1.6) was measured using spectrophotometry by the method of Chen and Wang [27]. The activity of nitrate reductase (NR, EC 1.7.1.1) was measured according to Jaworski [29].

#### 2.2.3. Measurement of Non-Enzymatic Antioxidants

Ascorbic acid (AsA) was determined by the method of Helrich [30], while glutathione content (GSH) was measured according to Griffith [31]. Total phenolic content (TPC) was extracted and analyzed according to Diaz and Martin [32]. Total flavonoid content was determined using the aluminum chloride colorimetric method [33]. Lycopene (Lyco) and β-carotene (β-car) were measured as described previously [34]. The free radical scavenging activity (DPPH%) was measured in plant extracts [35], while the 2,2′-azino-bis (3-ethylbenzothiazoline-6-sulfonic acid) enzymatic assay (ABTS%) for measuring ABTS cation radicals was performed according to the method described by Nenadis et al. [36].

### 2.3. Statistical Analysis

Data were subjected to an analysis of variance for a factorial experiment in a completely randomized design [37], after testing for the homogeneity of error variances using the Levene’s test [38], and after testing for normality distribution [39]. The Tukey’s HSD (honestly significant difference) test was used to compare the differences between means at *p* ≤ 0.05. The statistical analysis was conducted using GenStat 19th Edition (VSN International Ltd., Hemel Hempstead, UK).

## 3. Results

### 3.1. Changes in Photosynthetic Pigments and Endogenous IAA

Generally, photosynthetic pigments (chlorophyll a, chlorophyll b, chlorophyll a: chlorophyll b ratio, and total pigments) in addition to indole acetic acid contents were significantly reduced in wheat leaf tissue grown under salinity stress, while carotenoid contents were induced significantly compared with normal irrigated control plants (Table 1). Those decreases were recorded at 18.3, 11.6, 7.9, 10.5, and 31.4% under 30 mM NaCl and 38.1, 27.0, 15.0%, 26.3 and 49.6% under 60 mM NaCl for chlorophyll a, chlorophyll b, chlorophyll a: chlorophyll b ratio and total pigments in addition to indole acetic acid contents respectively, compared to unstressed plants. Carotenoid contents recorded 3.2% and 3.7% increases in response to the two studied salt concentrations in contrast to unstressed ones. Conversely, wheat plant treatment by Ca^2+^ (2.5 or 5.0 mM) induced significant increments in different photosynthetic pigment constituents under fresh irrigation water in comparison to untreated plants. It was revealed that foliar spraying of Ca^2+^ (2.5 or 5.0 mM) can reduce salinity’s harmful influence on photosynthetic pigments and indole acetic pigments contents compared to controls under two concentrations of salinity (30 or 60 mM NaCl). More increments of carotenoid concentration were obtained from wheat leaves that received two concentrations of Ca^2+^. It is clear from Table 1 that a higher concentration of Ca^2+^ was more effective than a lower one in increments of all the above-mentioned characters.

### 3.2. Changes in Osmoprotectants and Total Carbohydrates

Calcium affects the osmoprotectants accumulation i.e., free amino acids (FAA), proline (Pro), and total soluble sugars (TSS), as major osmolytes and total carbohydrates (CHO) in wheat plants in response to NaCl (Table 2). Subjecting wheat plants under two concentrations of NaCl 30 and (or) 60 mM increased significantly FAA, Pro, and TSS while significantly reducing CHO levels in comparison with controls. These increases were gradual with increasing salt stress concentrations. Furthermore, foliar spraying with Ca^2+^ (2.5 or 5.0 mM) improved significantly FAA, Pro, TSS as well as total carbohydrates contents of wheat plants. Concerning the effect of Ca^2+^ foliar treatment (2.5 and 5.0 mM) under different salt irrigation concentrations, Ca^2+^ caused more significant increments in different osmoprotectants and total carbohydrates than in untreated plants. According to the data in Table 2, higher (5.0 mM) Ca^2+^ concentrations are preferable to lower ones when it comes to boosting FAA, Pro, TSS, and TC under various saline irrigation concentrations.

### 3.3. Changes in Hydrogen Peroxide, Lipid Peroxidation, and Malondialdehyde

The impact of Ca^2+^ treatment on hydrogen peroxide (H_2_O_2_) and lipid peroxidation expressed by malondialdehyde (MDA) and aldehyde (ALD) under the impact of salt is shown in Table 3. Irrigation with different concentrations of saline water (30 or 60 mM NaCl) resulted in a rise in H_2_O_2_, MDA, and ALD compared to control plants, irrigated with distilled water. Those increases were gradually increasing with increasing salinity concentrations.

The increases in H_2_O_2_, MDA, and ALD were by 45.9, 28.3, and 55.5%, respectively, in plants irrigated with 60 mM NaCl in comparison with control ones. Lower H_2_O_2_, MDA, and ALD contents were obtained in wheat plants foliar sprayed with Ca (2.5 or 5.0 mM) in comparison with a control plant, except MDA contents, which showed marked increases with Ca^2+^ foliar application. Regarding the interaction impact between Ca^2+^ treatments and salt stress on H_2_O_2_, MDA, and ALD, different Ca^2+^ levels (2.5 or 5.0 mM) caused marked decreases in the above-mentioned characteristics except in MDA content, while 5.0 mM CaCO_3_ under distilled water irrigation and 2.5 or 5.0 mM Ca under 60 mM NaCl caused marked increases compared to the untreated ones.

### 3.4. Changes in Antioxidant Enzymes

The activity of several antioxidant enzymes viz superoxide dismutase (SOD), peroxidase (POD), catalase (CAT), and nitrate reductase (NR) in wheat leaves treated with calcium carbonate (Ca^2+^) and grown under the various NaCl concentrations are shown in Table 4. Different salt concentrations induced significant increments in SOD, POD, CAT and NR enzyme activities compared to the control plant. In wheat plants, 60 mM NaCl resulted in an increase by 82.9, 50.2, 70.8, and 44.3% in SOD, POD, CAT, and NR, respectively, compared to distilled water irrigated plants. Furthermore, different concentrations (2.5 or 5.0 mM) of calcium carbonate (Ca^2+^) treatment caused gradual and significant increments in the activity of the enzymes compared to the untreated control. An increase of 26.8, 23.3, 31.7, and 4.5% in SOD, POD, CAT, and NR, respectively, in wheat treated with 5.0 mM CaCO_3_ compared to the control was reported. Regarding the interaction impact of Ca^2+^ treatments and different concentrations of saline irrigation water, there was a significant increase in different studied enzymes (SOD, POD, CAT, and NR). Treatment with a high concentration of Ca^2+^ (5.0 mM) was more efficient in improving the activities of the studied enzyme under two salt concentrations compared to either control or application of 2.5 mM Ca^2+^.

### 3.5. Changes in Non-Enzymatic Antioxidants

Table 5 shows the impact of CaCO_3_ (Ca^2+^) on non-enzymatic antioxidants viz ascorbic acid (AsA), glutathione (GSH), total phenolic content (TPC), total flavonoid contents (TFC), β-carotene (β-car), and lycopene (Lyc) of wheat leaves grown under three concentrations of NaCl stress. Growing wheat plants under salt stress (30 or 60 mM NaCl) significantly increased AsA, GSH, TPC, and TFC, while reducing β-car and Lyc contents compared to control plants. The higher concentration of NaCl reduced ASA by 40.3%, GSH by 35.0%, TPC by 82.0%, and TFC by 81.4%, and increased β-car by 12.1% and Lyc by 17.1% compared to control unstressed plants. Exogenous treatment of Ca^2+^ with different concentrations (2.5 or 5.0 mM) induced significant increases not only in AsA, GSH, TPC, and TFC, but also in β-car and Lyc contents compared to untreated ones (0.0 mM). The higher concentration (5.0 mM) of Ca^2+^ was superior over the lower one (2.5 mM) in increasing AsA, GSH, and TPC, while the lower one (2.5 mM) was superior in increasing TFC, β-car, and Lyc contents. Regarding the interaction influence, it shows a significant effect on most of the above-mentioned parameters.

### 3.6. Changes in the Activity of Free Radical Scavenging and Enzymatic Scavenging

Irrigation of wheat plant either with 30 or 60 mM NaCl significantly increased 2,2-diphenyl-1-picrylhydrazyl-free radical scavenging assay (DPPH%) and 2,2′-azinobis (3-ethylbenzothiazoline-6-sulfonic acid) enzymatic assay (ABTS%) compared to control ones (Table 6). An increase in DPPH% by 21.6% while a 35.8% increase in ABTS% were observed in response to 60 mM NaCl compared to control plants. When compared to untreated ones, foliar application of CaCO_3_ (2.5 or 5.0 mM) caused a considerable rise in DPPH% and ABTS%. Foliar application of 2.5 mM Ca^2+^ resulted in a 14.6% increase in DPPH% and a 25.5% increase in ABTS%, which was the result of the 2.5 mM treatment. In terms of DPPH% and ABTS%, the interaction influence was considerable. Foliar spraying with Ca^2+^ induced significant increments of DPPH and ABTS of wheat plants over irrigation and it caused more significant increases compared to their corresponding controls (Table 6).

### 3.7. Changes in Plant Growth

Irrigation of wheat plants with 30 or 60 mM NaCl resulted in a significant gradual decrease in shoot length (SL), total leaf area per plant (LA), and shoot dry weight (SDW) compared to the unstressed control plant (Table 7). Foliar application of CaCO_3_ with 2.5 mM or 5.0 mM induced significant and also gradual increases in SL, LA, and SDW compared to untreated control. Regarding the impact of Ca^2+^ interaction with different salinity concentrations, wheat plants irrigated with 30 or 60 mM NaCl, Ca^2+^ exogenous treatments could improve markedly all studied wheat growth parameters viz. shoot length, total leaf area, and shoot dry weight (Table 7).

### 3.8. Treatment x Traits (TT) Biplot

**Figure 1:**
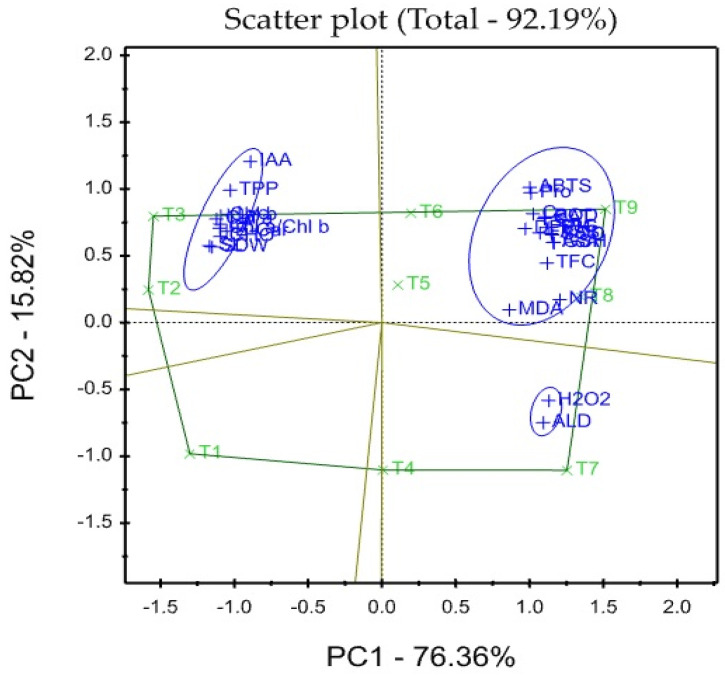
Polygon-view of TT biplot showing which factorial treatments of saline irrigation and Calcium had the highest values for wheat traits.

**Figure 2:**
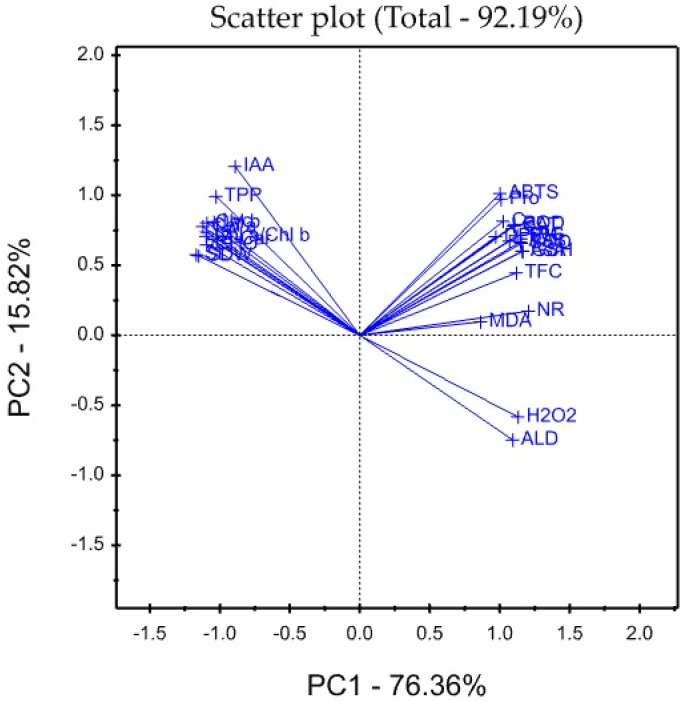
Vector view of TT biplot showing the interrelationship among measured traits of the wheat plant.

The mean values of the effects of two saline irrigation treatments and two calcium concentrations as foliar applications on the studied traits were graphically summarized in a polygon view, as shown in Figure 1. In addition, Figure 2 shows the vector view of the TT biplot showing the interrelationship among the measured traits of the wheat plant. The polygon view of the TT biplot distinguishes the factorial treatments of saline irrigation and foliar sprayed Ca, which gave the highest values for one or more traits. These findings show that the eigenvalues of the first three main components are greater than 1. The first and second main components (PC) accounted for 76.36 and 15.82% of the total explanation, respectively. Thus, the TT biplot-based principal components (PC) analysis explained roughly 92.19% of the observed variation for the assessed characteristics of wheat across saline irrigation and Ca foliar spray treatments (Figure 1). The first three principal components account for 95.20% of the data variation, whereas the first four principal components account for 97.00%.

These results indicated that the two biplot graphs were characterized by a goodness of fit model where the two biplot graphs can explain sufficient amounts of the total variation of more than 92.19% of the treatment x trait pattern data. There was a positive significant association between SDW and each of Chl a, Chl b, Chl a/Chl b, TPP, IAA, CHO, β-car, Lyc, SL, and LA, while there was a significant negative association between SDW and Car, FAA, Pro, TSS, H_2_O_2_, MDA, ALD, SOD, POD, CAT, NR, AsA, GSH, TPC, TFC, DPPH, and ABTS. The polygon graph shows the association between Ca and saline in the irrigation treatment combination considering wheat traits. The variables SDW, SL, and LA behaved better under T2 and T3, while H_2_O_2_ and ALD behaved better under T4 and T7.

## 4. Discussion

Salinity is one of the worst abiotic stressors that results in a growth rate slowdown, as well as several metabolic variations of plant metabolism [40,41]. Salt tolerance in plants is a genetic characteristic that differs between species, as observed considering the growth response [42]. Therefore, salt accumulation in plant cells might negatively impact regular physiological activities [43]. Consequently, plants evolved many quick strategies to mitigate various harms caused by salinity [44]. Additionally, a plant’s ability to modify harmful environmental stresses is improved via the escape methods and the presence of tiny substances including phytohormones and signaling molecules. This investigation revealed that foliar application of Ca modifies the cell osmo-protection and free radical scavenging capacity to modify growth and development in salinity conditions.

The presented data in Table 1 clearly show that photosynthetic pigment contents were decreased under salinity stress. These decreases in several photosynthetic pigment constituents were confirmed in various plants such as the common bean [45] and wheat [46]. Those decrements could be attributed to increased levels of oxidative stress and the generation of free radicals owing to salt stress, which harms chloroplast membranes, as well as the antagonism of sodium ions on magnesium ion uptake [47]. Additionally, those reductions might be seen as a crucial regulator step to prevent high light uptake and as a strategy for reducing excessive decreases in the electron transport chain, which would enhance free radical accumulation, thereby resulting in increased production of reactive oxygen species (ROS) and oxidative stress owing to salinity stress, and thus damaging chloroplast membranes and resulting in antagonistic effects of sodium ion on magnesium ion absorption [48]. Moreover, increased salinity concentrations resulted in an increment in Na^+^ and Cl^−^ ions deposition, which interfere with the process of chlorophyll formation, by altering the activity of chlorophyll synthesizing enzymes containing Fe^3+^ [49] or by increasing the activity of chlorophyllase and excess accumulation of ROS [50]. Furthermore, salt stress caused improved proline biosynthesis, which causes significantly less glutamate usage as a precursor in biosynthesizing chlorophyll molecules [49]. Meanwhile, carotenoid content increased owing to salt stress; these elevated carotenoid contents could be reflected in the impact of carotenoids for free radical scavenging. Therefore, carotenoids help in increasing a plant’s ability in lessening the harm from free radicals, thus improving chlorophyll a and chlorophyll b concentrations of wheat leaves tissue. This enhancement gained by Ca foliar spraying of the wheat plant was similar to that observed in pepper [51], tung tree [52], and wheat [53]. These improvements could be due to the role of Ca^2+^ in minimizing dehydration injury of cellular structures by regulating the osmotic strength of cytoplasm in plants [54]. The application of Ca has an impact on the majority of physiological processes in salt-stressed plants [55], and our results supported this.

Abiotic stressors such as salt affect diverse plant hormone contents, causing an increase in growth inhibitors and a decrease in growth promoters such as auxins. The most common natural auxin indole acetic acid (IAA) is a plant growth regulator. Indole acetic acid is very effective in controlling the growth and development of different crops [56]. The reduced IAA contents of wheat plants in saline conditions (Table 1) were associated with the decline in growth attributes (Table 7). Reduced IAA contents under salt stress may be related to increased breakdown, its transformation to inactive form, or inhibition of IAA production [57].

With tap and saline water, foliar Ca application led to appreciable increases in endogenous IAA. It was proved that Ca promotes IAA formation in the wheat plant [53]. Those increments could be the result of Ca’s improved impact in hastened IAA formation, transportation, and decreased IAA oxidase activity [53].

Osmolyte production is a typical sight in stress mitigation to lessen physiological harm. This research stated that wheat plants deposit extra proline in salty conditions. The obtained data have been confirmed in various plant species under stress, for example, in the common bean [45] and wheat [46]. In addition to being essential for regulating osmotic balance, proline is used as a molecular chaperone to prevent membrane damage [58]. Proline also functions as an antioxidant, preventing oxidative damage to cellular structures and different enzymes, and aids in scavenging accumulated ROS. The overproduction of free amino acids might be the cause of proteolysis during osmotic adjustment. Thus, FAA build-up might be an adaptive method for plants to control cell osmotic potential, thereby improving water absorption and transportation under salty conditions [47]. Herein, in our study, Ca with different concentrations significantly increased FAA, Pro, and TSS levels of wheat leaves. The data of FAA, Pro, and TSS were confirmed in earlier studies [53], as it was shown before.

Regarding the carbohydrate levels of wheat plants subjected to different saline water concentrations, salt decreased the carbohydrate contents (CHO) (Table 2). This reduction was in parallel with the decrease in total photosynthetic pigments (TPP) (Table 1). Carbohydrate changes in wheat leaves are extremely meaningful due to their close connection to physiological functions. On the contrary, calcium carbonate application enhanced CHO concentration, which may improve growth parameters and TPP (Table 1 and Table 2) [53].

Salt stress induces over-accumulation of free radicals in plant cells; these ROS affect adversely various chemical structures and performance of enzyme, nucleic acids, and lipids, as well as other essential components. A prominent symptom of stress-related cell damage is membrane peroxidation caused by free radicals [59]. Although H_2_O_2_, MDA, and ALD are essential for plant metabolism, their overproduction is detrimental, particularly towards membranes. It was found that higher levels of H_2_O_2_, MDA, and ALD could lead to electrolyte leakage, a sign of deteriorating membrane integrity [44,55]. Additionally, the insufficient induction of the antioxidant system may be the cause of the stress impact on MDA and H_2_O_2_. Several metabolic activities and signaling cascades necessary for plant growth and development utilize hydrogen peroxide. The interaction of the two factors (saline irrigation and calcium foliar spray) ultimately showed that Ca plays a significant role in reducing the effects of salt stress in wheat plants by altering membrane integrity, cell wall structure, and enhancing the plant’s water status or by directly acting on osmolytes; H_2_O_2_ is used in various biochemical processes and signaling cascades necessary for plant growth and development. Generally, H_2_O_2_ contents increased in various plants subjected to stress [6,60]. Calcium carbonate also reacts with phytohormones and is used in cell wall formation [14,61]. It was notable that Ca uses successfully reduced lipid peroxidation by decreasing H_2_O_2_, MDA, and ALD concentrations.

Antioxidants, either antioxidant enzymes or antioxidant compounds, have a very important impact on improving plant tolerance against accumulated reactive oxygen species (ROS). Herein, salinity stress increased antioxidant enzyme activity (POD, SOD, CAT, and NR) in comparison with unstressed plants. The salinity-promoting impact has been reported previously [6,60,62]. Oxidative stress is a key indicator of abiotic stress, and the increased POD, SOD, CAT, and NR activities were associated with increased protection from the destruction caused by oxidative stress [60]. Peroxidases are essential enzymes in many physiological pathways in the plant, under abiotic stresses. In addition, it has a role as a free radical scavenger and cell protector against H_2_O_2_ damage [25]. Superoxide dismutase transforms superoxide radicals into H_2_O_2_ (that remains hazardous) and needs to be eliminated by being transformed to H_2_O in the following step. Peroxidase then converts H_2_O_2_ to H_2_O [63]. Moreover, H_2_O_2_ could be eliminated from the cell via CAT by reducing H_2_O_2_ to oxygen and water, resulting in improved plant tolerance. Hydrogen peroxide outside chloroplasts is effectively removed by POX in the cytosol and peroxisomes [64]. Owing to peroxidase’s dependence on Ca and functional inactivity in the absence of Ca, the foliar application of Ca enhanced POX activity, as Ca plays a cofactor in POX [65]. Nitrate reductase (NR) increased with increased levels of salinity stress (Table 4). The increased activities of the NR enzyme resulted in an increased level of FAA contents [66]. Moreover, other researchers confirmed our data [16].

Antioxidant compounds of the wheat plant under stress include AsA, GSH, TPC, and TFC in addition to non-photosynthetic pigments such as β-car and Lyc. By subjecting the plant to abiotic stress such as salinity, certain regulating substances were found to be overproduced under stress [67]. Increased AsA and GSH production in response to salt stress might have significantly contributed to salinity tolerance via (a) stabilizing redox homeostasis, (b) scavenging ROS, and (c) preserving the activity of the ascorbate–glutathione cycle including the ascorbate peroxidase and GSH. Additionally, GSH plays a crucial signaling role in shielding photosynthetic pigments from stress brought on by oxidative conditions [68]. Increased GSH production in conjunction with glutathione reductase (GR) up-regulation is important for maintaining NADP levels for steady photosynthetic electron transport.

Phenolic (TPC) and flavonoids (TFC) are among the antioxidants substances that initiate a series of secondary metabolites produced by shikimic acid or malonic acid cycles, and it serves as cell signaling agents [69]. Similar results were obtained earlier for different plant species, for example, in wheat plants [6]. Phenols are known as antioxidant substances that support the defense system and scavenge ROS brought on by various stressors. An increment in phenol amounts could modulate the negative influence of salt stress [70]. Plants create more phenolic compounds as a result of physiological changes brought on by stress [71]. Increased free radicals are usually associated with shifts in net carbon uptake in the plant, and these alterations have a substantial influence on the signaling pathways of secondary substances, particularly polyphenols [72]. Additionally, phenols are essential antioxidants and free radical scavengers [71]. The antioxidant action of flavonoids is dependent on the availability of free OH groups [73]. Flavonoid-improved levels under salinity might be a form of defense (i.e., oxidative burden). The impact of Ca on TPC is important according to Ma et al. [74]. They stated that the application of Ca significantly enhanced phenolic concentrations and enzymatic activity utilized in phenol metabolism (phenylalanine ammonia-lyase, polyphenol oxidase, and peroxidases). Furthermore, the increased TPC might be referred to as improved phenylalanine ammonia-lyase activity, which is crucial for phenol production [74]. Spraying wheat plants with Ca caused increases in flavonoid concentrations. Those findings suggest the Ca can stimulate the production of secondary metabolites (TFC) that function as oxygen scavengers to minimize oxidative stress and thus boost wheat growth and yield (Table 5).

β-car and lyc are non-photosynthetic pigments; however, lyc is a precursor of β-car, a fat-soluble carotenoid with two times as much as antioxidation activity than β-car. Lycopene’s possible antioxidant properties are mostly related to conjugated double bond that can quench free radicals [75]. Lyc also plays crucial roles in metabolic pathway, hormone, and immunological response regulation, as well as cell signaling and connection [76]. Of the prominent natural antioxidants, lyc, and β-car operate as the most efficient singlet oxygen quenchers. Because Car is a component of the mechanism that absorbs sunlight, Car has a close relationship with the photosynthetic mechanism [77].

Salinity increased the antioxidant activity (DPPH%) and ABT% of a wheat plant. It was stated that increased antioxidant activities and phenol content were obtained in wheat and wheat-based food products, indicating that wheat may serve as an excellent dietary source of natural antioxidants for disease prevention and health promotion [78]. The increase in the scavenging activity can be considered an advantage of the treatment used. This could be attributed to the increases in total phenols and total flavonoids [79].

As a first indication, reduction in plant growth is the main feature of salinity [57,80,81]. Other authors on wheat plants have lately validated these acquired results [6]. Herein, in this investigation, salt stress reduced wheat growth; this effect resulted from carbon fixation reduction due to specific ion toxicity [82]; partial stomata closure that caused the reduction of photosynthesis, reduced energy in osmotic adaptation, and ion exclusion [83,84]; and nutritional imbalances [85]. Plants under high salt concentrations encounter great disorders such as hyperionic, hyperosmotic, and ionic disorders that disrupt different metabolic processes, therefore decreasing plant yield. For calcium carbonate treatment, the enhancement effect of Ca on the growth criteria compared with the control plant confirmed the important role of Ca in the alleviation of salinity stress of growth and yield of the wheat plant. Similar findings were reported earlier, concurrent with our obtained results of Ca on the different plants under abiotic stresses [6,53]. The positive role of Ca on growth might be attributed to the fact that Ca was a crucial secondary messenger that is used in signaling-related processes to many defense mechanisms that are induced by salinity stress [86]. All growth parameters, including shoot length, leaf area, and dry weights, were drastically decreased due to either salt stress or without Ca foliar application, both alone or when they were combined (Table 7). A decrease in calcium availability inhibits plant growth because calcium is necessary for maintaining the integrity of cell walls and membranes. A drop in the Ca^2+^ concentration of the saline solution may further inhibit plant growth. It might be caused by the loss of Ca^2+^ ions from the internal pool and cell plasma membrane [87]. A combined stress of low calcium and salt reduced all growth metrics more than a stress of salinity alone or low calcium alone, in that order.

Ca can increase membrane stability and protect them from lipid peroxidation and oxidative stress induced by salinity stress, thus improving the water status of plants [88], as calcium is an important constituent of the plant cell wall and plays an important role in cell division and enlargement. Sodium is the predominant cation under saline conditions caused by the flux of Na^+^, which competes with Ca at the binding sites. External treatment with Ca could alleviate the destructive consequences via protection of the integrity and plasma membrane permeability towards Na^+^ toxicity [89]. External treatment of calcium chloride first alters Na^+^ influx and maintains Na^+^ and K^+^ homeostasis [90].

## 5. Conclusions

The antioxidant system including antioxidant enzymes and non-enzymatic antioxidants was upregulated by Ca application, resulting in a reduction in reactive oxygen species accumulation (ROS). Under salt or non-salt stress conditions, reduced ROS in Ca-treated plants resulted in decreased hydrogen peroxide, lipid peroxidation (MDA), and aldehyde levels. Moreover, Ca foliar spray increased compatible solute accumulation (free amino acids, proline, total soluble sugar, and carbohydrate) as well as DPPH% and enzymatic ABTS% radical scavenging activity. Salt stress reduced photosynthetic pigments, endogenous indole-3-acetic acid (IAA), shoot length, total leaf area, and shoot dry weight of the wheat plant. Moreover, lycopene content decreased with NaCl stress. In contrast, foliar application of Ca improved photosynthetic pigments and endogenous indole-3-acetic acid, which enhanced plant leaf area and, as a result, increased biomass dry weight in both salt-stressed and non-stressed plants.

## Figures and Tables

**Table 1:** Effect of Ca foliar application on chlorophyll a (Chl a), chlorophyll b (Chl b), Chl a: Chl b ratio, carotenoids (Car), total photosynthetic pigments (TPP), and indoleacetic acid (IAA) in wheat plants grown under salt stress (NaCl) for 60 days.

Treatment	Ca (mM)	Chl a	Chl b	Chl a/Chl b	Car	TPP	IAA
		(mg g^−1^ FW)					(μg g^−1^ FW)
NaCl (mM)							
0.0		2.02 ^†^ a	0.95 a	2.13 a	0.38 c	3.34 a	9.40 a
30		1.65 b	0.84 b	1.96 b	0.50 b	2.99 b	6.44 b
60		1.25 c	0.69 c	1.81 c	0.52 a	2.46 c	4.74 c
Ca (mM)							
0.0		1.45 c	0.76 c	1.90 c	0.42 c	2.63 c	4.68 c
2.5		1.67 b	0.84 b	1.97 b	0.46 b	2.98 b	6.93 b
5.0		1.79 a	0.88 a	2.03 a	0.51 a	3.17 a	8.98 a
NaCl (mM)	Ca (mM)						
0	0.0	1.80 c	0.86 c	2.08 a	0.35 e	3.01 d	6.50 d
	2.5	2.07 b	0.96 b	2.15 a	0.36 e	3.39 b	9.28 b
	5.0	2.18 a	1.01 a	2.15 a	0.41 d	3.61 a	12.41 a
30	0.0	1.49 e	0.79 d	1.88 b	0.42 d	2.70 e	4.34 f
	2.5	1.67 d	0.87 c	1.92 b	0.51 b	3.05 d	6.55 d
	5.0	1.78 c	0.86 c	2.08 a	0.56 a	3.21 c	8.44 c
60	0.0	1.07 h	0.62 g	1.73 c	0.49 c	2.18 g	3.18 g
	2.5	1.28 g	0.69 f	1.85 b	0.52 b	2.50 f	4.95 e
	5.0	1.39 f	0.75 e	1.85 b	0.55 a	2.69 e	6.09 d

^†^ Mean values within the same column for each trait with the same lower-case letter are not significantly different according to Tukey’s honestly significant difference (HSD) test at *p* ≤ 0.05. The results presented are from wheat plants harvested 75 days after sowing (DAS).

**Table 2:** Effect of Ca foliar application on compatible solute accumulation (free amino acids; FAA, proline; Pro, total soluble sugar; TSS) and carbohydrates (CHO) in wheat plants grown under salt stress (NaCl) for 60 days.

Treatment	Ca (mM)	FAA	Pro	TSS	CHO
			(mg g^−1^ FW)		(%)
NaCl (mM)					
0.0		235 ^†^ c	34.3 c	44.6 c	45.6 a
30		265 b	43.6 b	63.7 b	44.1 b
60		325 a	59.0 a	82.9 a	42.1 c
Ca (mM)					
0.0		253 c	37.2 c	55.6 c	43.0 c
2.5		281 b	45.6 b	64.0 b	44.8 a
5.0		291 a	54.3 a	71.7 a	44.0 b
NaCl (mM)	Ca (mM)				
0	0.0	225 i	29.2 i	41.7 g	44.3 d
	2.5	237 h	34.1 h	42.8 g	46.8 a
	5.0	241 g	39.7 f	49.4 f	45.8 b
30	0.0	254 f	35.9 g	51.9 e	43.2 e
	2.5	274 d	43.9 e	66.3 d	44.9 c
	5.0	268 e	51.1 c	72.9 c	44.1 d
60	0.0	279 c	46.4 d	73.2 c	41.4 g
	2.5	330 b	58.7 b	82.9 b	42.7 ef
	5.0	365 a	72.0 a	92.7 a	42.2 f

^†^ Mean values within the same column for each trait with the same lower-case letter are not significantly different according to the Tukey’s honestly significant difference (HSD) test at *p* ≤ 0.05. The results presented are from wheat plants harvested 75 days after sowing (DAS).

**Table 3:** Effect of Ca foliar application on hydrogen peroxide (H_2_O_2_), lipid peroxidation (MDA), and aldehyde (ALD) in wheat plants grown under salt stress (NaCl) for 60 days.

Treatment	Ca (mM)	H_2_O_2_	MDA	ALD
		(nmol g g^−1^ FW)		
NaCl (mM)				
0.0		0.222 ^†^ c	0.456 b	0.220 c
30		0.296 b	0.556 a	0.292 b
60		0.324 a	0.585 a	0.342 a
Ca (mM)				
0.0		0.297 a	0.517 a	0.314 a
2.5		0.280 b	0.558 a	0.275 b
5.0		0.264 c	0.522 a	0.264 c
NaCl (mM)	Ca (mM)			
0	0.0	0.231 e	0.449 b	0.235 e
	2.5	0.223 e	0.434 b	0.213 f
	5.0	0.212 e	0.487 ab	0.212 f
30	0.0	0.320 b	0.605 ab	0.317 b
	2.5	0.296 c	0.552 ab	0.289 c
	5.0	0.273 d	0.512 ab	0.270 d
60	0.0	0.342 a	0.499 ab	0.390 a
	2.5	0.321 ab	0.688 a	0.324 b
	5.0	0.308 bc	0.568 ab	0.311 b

^†^ Mean values within the same column for each trait with the same lower-case letter are not significantly different according to the Tukey’s honestly significant difference (HSD) test at *p* ≤ 0.05. The results presented are from wheat plants harvested 75 days after sowing (DAS).

**Table 4:** Effect of Ca foliar application on enzymatic antioxidants (superoxide dismutase; SOD, peroxidase; POX, catalase; CAT, and nitrate reductase; NR) in wheat plants grown under salt stress (NaCl) for 60 days.

Treatment	NaCl (mM)	SOD	POD	CAT	NR
		(U/min/g FW)			(nM NO_2_ g^−1^ FW)
NaCl (mM)					
0.0		31.5 ^†^ c	62.1 c	38.3 c	323 c
30		47.0 b	78.5 b	54.4 b	378 b
60		57.6 a	93.3 a	65.4 a	466 a
Ca (mM)					
0.0		39.6 c	69.9 c	46.1 c	380 c
2.5		46.2 b	77.9 b	51.3 b	390 b
5.0		50.2 a	86.2 a	60.7 a	397 a
NaCl (mM)	Ca (mM)				
0	0.0	27.9 h	57.9 i	33.7 h	324 g
	2.5	30.4 g	62.6 h	38.0 g	330 g
	5.0	36.0 f	65.9 g	43.0 f	316 h
30	0.0	39.9 e	67.8 f	47.4 e	363 f
	2.5	49.3 d	77.9 e	52.9 d	377 e
	5.0	51.9 c	89.9 c	62.8 b	393 d
60	0.0	51.1 c	84.1 d	57.1 c	454 c
	2.5	58.9 b	93.1 b	62.9 b	463 b
	5.0	62.8 a	102.9 a	76.3 a	481 a

^†^ Mean values within the same column for each trait with the same lower-case letter are not significantly different according to the Tukey’s honestly significant difference (HSD) test at *p* ≤ 0.05. The results presented are from wheat plants harvested 75 days after sowing (DAS).

**Table 5:** Effect of Ca foliar application on non-enzymatic antioxidants namely ascorbic acid (AsA), glutathione (GSH), total phenolic content (TPC), total flavonoid content (TFC), β-carotene (β-car) and lycopene (Lyc) in wheat plants grown under salt stress (NaCl) for 60 days.

Treatment	Ca (mM)	AsA	GSH	TPC	TFC	β-Car	Lyc
		(µmol ASA/100 g DW)	(µmol GSH/100 g DW)	(mg/100 g DW)			
NaCl (mM)							
0.0		238 ^†^ c	137 c	48.9 c	11.8 c	0.405 a	0.362 a
30		292 b	162 b	77.2 b	16.5 b	0.374 b	0.326 b
60		334 a	185 a	89.0 a	21.4 a	0.356 c	0.300 c
Ca (mM)							
0.0		269 c	152 c	62.1 c	14.6 c	0.363 c	0.308 c
2.5		290 b	161 b	72.4 b	18.6 a	0.394 a	0.347 a
5.0		305 a	170 a	80.6 a	16.6 b	0.379 b	0.334 b
NaCl (mM)	Ca (mM)						
0	0.0	219 i	130 i	42.9 g	11.2 f	0.383 cd	0.336 c
	2.5	240 h	137 h	48.7 f	12.3 ef	0.426 a	0.376 a
	5.0	256 g	144 g	55.0 e	12.0 f	0.407 ab	0.375 a
30	0.0	268 f	149 f	64.4 d	13.9 e	0.362 def	0.307 e
	2.5	294 e	163 e	79.2 c	19.6 c	0.389 bc	0.347 b
	5.0	314 d	173 d	88.1 b	15.9 d	0.372 cde	0.323 d
60	0.0	321 c	177 c	79.0 c	18.5 c	0.345 f	0.281 f
	2.5	335 b	184 b	89.2 b	24.0 a	0.366 def	0.317 d
	5.0	347 a	194 a	98.7 a	21.8 b	0.358 ef	0.303 e

^†^ Mean values within the same column for each trait with the same lower-case letter are not significantly different according to the Tukey’s honestly significant difference (HSD) test at *p* ≤ 0.05. The results presented are from wheat plants harvested 75 days after sowing (DAS).

**Table 6:** Effect of Ca foliar application on 2,2-diphenyl-1-picrylhydrazyl-free radical scavenging assay (DPPH%) and 2,2′-azinobis (3-ethylbenzothiazoline-6-sulfonic acid) enzymatic assay (ABTS%) in wheat plants grown under salt stress (NaCl) for 60 days.

Treatment	Ca (mM)	DPPH (%)	ABTS (%)
NaCl (mM)			
0.0		57.8 ^†^ c	36.0 c
30		64.5 b	44.6 b
60		70.3 a	48.9 a
Ca (mM)			
0.0		59.7 c	38.5 c
2.5		68.4 a	42.6 b
5.0		64.6 b	48.3 a
NaCl (mM)	Ca (mM)		
0	0.0	51.4 f	33.1 h
	2.5	64.1 d	35.1 g
	5.0	58.0 e	39.7 f
30	0.0	59.8 e	38.8 f
	2.5	68.0 bc	45.2 d
	5.0	65.8 d	49.8 b
60	0.0	67.9 c	43.8 e
	2.5	73.2 a	47.4 c
	5.0	70.0 b	55.4 a

^†^ Mean values within the same column for each trait with the same lower-case letter are not significantly different according to the Tukey’s honestly significant difference (HSD) test at *p* ≤ 0.05. The results presented are from wheat plants harvested 75 days after sowing (DAS).

**Table 7:** Effect of Ca foliar application on shoot length (SL), total leaf area per plant (LA), and shoot dry weight per plant (SDW) in wheat plants grown under salt stress (NaCl) for 60 days.

Treatment	Ca (mM)	SL	LA	SDW
		(cm)	(cm^2^ Plant^−1^)	(g Plant^−1^)
NaCl (mM)				
0.0		50.5 ^†^ a	182 a	1.98 a
30		43.8 b	112 b	1.17 b
60		36.5 c	88 c	0.76 c
Ca (mM)				
0.0		41.4 c	105 c	1.10 c
2.5		43.7 b	129 b	1.29 b
5.0		45.8 a	148 a	1.51 a
NaCl (mM	Ca (mM)			
0	0.0	47.6 bc	145 c	1.62 c
0	2.5	50.7 ab	187 b	1.93 b
0	5.0	53.3 a	214 a	2.38 a
30	0.0	42.2 de	95 de	1.05 e
30	2.5	43.8 cd	110 d	1.17 de
30	5.0	45.5 cd	131 c	1.28 d
30	0.0	34.3 g	76 f	0.64 g
30	2.5	36.5 fg	89 ef	0.76 fg
30	5.0	38.7 of	99 de	0.87 f

^†^ Mean values within the same column for each trait with the same lower-case letter are not significantly different according to the Tukey’s honestly significant difference (HSD) test at *p* ≤ 0.05. The results presented are from wheat plants harvested 75 days after sowing (DAS).

## Data Availability

The original contributions presented in the study are included in the article. Further inquiries can be directed to the corresponding author.
